# Panel survey data on the investment and risk behavior of Neobroker users in Germany

**DOI:** 10.1016/j.dib.2025.111775

**Published:** 2025-06-10

**Authors:** Jonas Freibauer, Marc Oliver Rieger, Silja Grawert

**Affiliations:** a University of Trier, Department of Economics, Universitätsring 15, 54296 Trier, Germany; b Munich University of Applied Sciences, Lothstraße 64, 80335 Munich, Germany

**Keywords:** Trading app, Investment behavior, Risk behavior, Longitudinal study, Germany

## Abstract

This paper presents data consisting of three waves of a longitudinal panel survey conducted among Neobroker users in Germany. The panel survey spans over 14 months with data collected in December 2022, August 2023, and February 2024. This staggered timing allows for the examination of both individual and collective longitudinal effects of trading apps on investment behavior by observing changes and trends over time. Each wave of the survey used filter questions to target specific groups, including current Neobroker users, former users, general online investors, and usage planners. The datasets provide insights into investment and risk behavior among diverse groups of participants, with a focus on Neobroker usage. The data facilitate the exploration of collective investment and risk behavior across these groups, highlighting potential trends and commonalities. The representative sampling methodology ensures reliability and reduces sampling bias. The datasets allow the analysis of trading app impact on user behavior over time, due to repeatedly surveyed Neobroker users. Repeated questioning also enables to identify reasons behind changes in trading app usage, such as why former Neobroker users stopped using Neobrokers during the survey waves. Additionally, they provide the opportunity to compare the characteristics and the behavior of Neobroker users with general online investors. By offering diverse participant insights, these datasets serve as a strong foundation for examining broader investment and risk patterns and the collective investment and risk behavior among different groups.

Specifications Table

**Table:** 

Subject	Social Sciences
Specific subject area	Impact of trading apps on the investment and risk behavior of Neobroker users
Type of data	Table, Supporting material (questionnaires), Raw data
Data collection	Data were collected by a company conducting representative online-surveys in Germany, which has its own panel partner to recruit respondents for panel-surveys. The research group established minimum respondent quotas for each group in the respective survey, with individuals under the age of 16 being excluded from participating in the surveys. The questionnaires were created by the research group, while the surveys were conducted by the survey company. The questionnaires included randomized answer options for suitable questions. To ensure the integrity of the data sets, a test question was incorporated into each questionnaire to eliminate the possibility of random and incorrect answers. Participants who provided an incorrect answer to this question were excluded from the respective survey. The survey questions derived from a pre-study and from previous academic literature (e.g. measuring of financial literacy).
Data source location	The data were collected in Germany. The datasets are stored at the University of Trier, Germany and the Munich University of Applied Sciences, Germany.
Data accessibility	Repository name: Panel survey data on the investment and risk behavior of Neobroker users in Germany Data identification number: doi: 10.17632/jv36jsfd8v.2 Direct URL to data: https://data.mendeley.com/datasets/jv36jsfd8v/2
Related research article	J. Freibauer, S. Grawert & M.O. Rieger, The effects of trading apps on investment behavior over time, The European Journal of Finance (2024). https://doi.org/10.1080/1351847X.2024.2401604

## 1 Value of the Data

• These data are useful in understanding the effects of trading app usage on the investment and risk behavior of Neobroker users over time.
• By eliminating sample biases through representative sampling, these datasets can be reused to validate predictions about the financial decision-making processes influenced by trading apps.
• The datasets provide representative data on Neobroker users in general, which means across different trading apps. Data used in previous studies about Neobroker users used data from one specific trading app [10] which could cause a sample bias.
• Researchers, social and public sector leaders, program managers, and policymakers can benefit from these datasets through its longitudinal setting with repeatedly (two or three times) surveyed Neobroker users over a period of 14 months and its setting to include general online investors and former Neobroker users.
• The datasets fill a gap in understanding general Neobroker usage beyond single platform studies and contribute valuable data to the broader discourse on FinTech influence.
• The data can be analyzed with univariate, bivariate, and multivariate methods.

## 2 Background

The motivation behind these dataset collections was to study the impact of trading apps on the investment and risk behavior of Neobrokers users as well as on the stability of financial markets. Prior studies on similar topics often relied on data from specific trading apps, a method that risks sample bias. Our datasets cover a diverse range of trading apps, providing a holistic view of the Neobroker landscape in Germany. This data article adds value to Freibauer et al. (2024) [1] by providing the scientific community with unique representative longitudinal data of Neobroker users and various demographic variables, which can especially be used for an intercultural and multinational analyses of the effects shown in this study, by replicating the datasets in different countries. Additionally, researchers will be able to use the datasets to conduct a variety of statistical analyses, which have not been shown in the paper [1], as for example the impact of low trading fees on the investment behavior of Neobroker users.

## 3 Data Description

The datasets comprise three waves of a panel survey, each collected at different time periods. The data of the three waves of the panel survey and the respective questionnaires are available through Mendeley Data [2]. The datasets consist of raw survey responses. Each dataset varies slightly, focusing on different user categories such as Neobroker users and general investors. Data are structured with variables corresponding to user engagement and investment behavior metrics. Dataset 1 reflects the first wave of the panel survey and consists of 502 participants in total (257 Neobroker users, 132 Neobroker usage planners, 113 Ex-Neobroker users). Dataset 2 presents the second wave of the panel survey and consists of 537 participants (246 Neobroker users, 122 Neobroker usage planners, 191 general online investors). Dataset 3 represents the third wave of the panel survey and consists of 524 participants (264 Neobroker users, 252 general online investors, 8 Ex-Neobroker users).

**Fig. 1:**
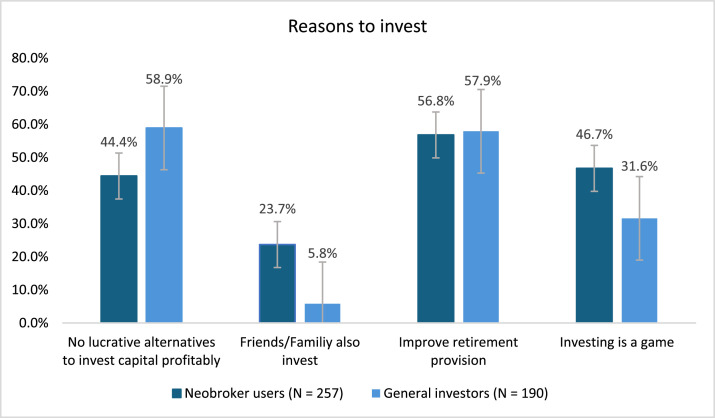
Reasons to invest for Neobroker users (data of the first dataset: N = 257) and general investors (data of the second dataset: N = 190). Please note that multiple answers were possible.

The comprehensive summaries in Table 1, Table 2, Table 3, Table 4 and Fig. 1 provide a clear view of the distinct perspectives and behaviors of each group regarding investment and risk behavior in the context of trading apps. Table 1 provides an overview on the composition of the respective datasets regarding the different target groups. There is no data from general investors in dataset 1 and no data from Neobroker usage planners in dataset 3, as the respective group was not part of the target groups of the respective survey.

**Table 1:** Overview of participant groups across the three survey waves (not free of overlaps).

Wave	Neobroker users (N)	Ex-Neobroker users (N)	Planners (N)	General investors(N)	Total participants
1	257	113	132	-	502
2	246	80	122	191	537
3	264	8	-	252	524

The respective survey questionnaire is available as pdf file. The file containing all three questionnaires is called “Questionnaires_Panel_survey_Neobroker_users”. This file includes the survey questions, target groups and the response options and shows restrictions for the general participation (e.g. age) and for specific questions (e.g. single / multiple choice possible). The file is available through Mendeley Data [2]. The questionnaires are designed to meet the respective target groups of each survey, so that there are several questions which needed to be phrased differently for the specific target groups. In this case, when the questions ask about the same topic, but needed to be rephrased, the questions are numbered accordingly (e.g. Question S10a, Question S10b, Question S10c). The respective question got only displayed to the respective target groups and therefore only answered by the respective target group.

The surveys were designed to understand the sociodemographic and investment behavior of the respondents (Modul S: QS1 – QS10), the trading app usage (Modul A: QA11 – QA16 and QS4), the broker selection (Modul W: QW17 – QW24), the financial knowledge and information behavior (Modul F: QF25 – QF29), and the characteristics and attitudes of former Neobroker users (Module E: QE30 – QE31). Descriptive analysis of many, but not all, of the findings of the surveys can be found in two published papers [1,6].

The data is meticulously organized into the three survey waves. Each dataset from the respective survey can be accessed individually via Mendeley Data [2]. All questionnaires are compiled into a single PDF document, which can also be accessed via Mendeley Data [2].. To ensure the unique identification of participants across the three survey waves, datasets 2 and 3 include the identification numbers of previous datasets of participants who were repeatedly surveyed. Table 2 shows the sample characteristics of each dataset and is accompanied by Table 3, which shows the personal information of all participants of each dataset.

**Table 2:** Sample characteristics of the various survey samples and waves.

Dataset	Time	Observation	Female	Diverse	Mean age*	University degree⁎⁎
**Dataset 1**		502	37.2%	0.2%	44	45.5%
**1 -** Neobroker users	08.-21. December 2022	257	37.0%	0.0%	40	54.1%
**1 -** Planners	08.-21. December 2022	132	45.5%	0.0%	47	34.1%
**1 -** Ex-Neobroker users	08.-21. December 2022	113	35.4%	0.9%	48	39.8%
**Dataset 2**		537	39.8%	0.0%	46	46.1%
**2 -** Neobroker users	08.-22. August 2023	246	37.0%	0.0%	41	51.2%
**2 -** General Investors	08.-22. August 2023	191	35.6%	0.0%	51	47.6%
**2 -** Planners	08.-22. August 2023	122	46.7%	0.0%	50	47.5%
**2 -** Ex-Neobroker users	08.-22. August 2023	80	43.8%	0.0%	48	38.8%
**Dataset 3**		524	42.7%	0.0%	46	40.6%
**3 -** Neobroker users	05.-20. February 2024	264	36.7%	0.0%	42	45.1%
**3 -** General Investors	05.-20. February 2024	252	50.0%	0.0%	50	35.3%
**3 -** Ex-Neobroker users	05.-20. February 2024	8	12.5%	0.0%	44	62.5%

⁎ “Mean age” for the respective dataset refers to the average age of the entire respective dataset. The “Mean age” of each subset refers to the average age of the respective group (e.g. the average age of Neobroker users in dataset 1 is 40).

⁎⁎ “University degree” for the respective dataset displays the respective proportion of participants who have obtained a university degree. “University degree” of each subset reflects the respective proportion of the subset which has a university degree.

**Table 3:** Personal information of the participants of each dataset.

Variables	Groups	Dataset 1 Number of participants (%)	Dataset 2 Number of participants (%)	Dataset 3 Number of participants (%)
Gender	Male	315 (62.8)	324 (60.3)	300 (57.3)
	Female	186 (37.0)	213 (39.7)	224 (42.7)
	Diverse	1 (0.2)	0 (0.0)	0 (0.0)
Age	17-26	42 (8.35)	36 (6.7)	30 (5.7)
	27-34	110 (21.87)	90 (16.7)	89 (17.0)
	35-48	162 (32.2)	178 (33.1)	174 (33.2)
	49-64	155 (30.82)	184 (34.2)	200 (38.2)
	> 64	34 (6.76)	50 (9.3)	31 (5.9)
School education	no school diploma	0 (0.0)	2 (0.4)	0 ()
	Hauptschule/Realschule diploma	162 (32.2)	166 (30.9)	178 (34.0)
	High school diploma (or equal)	341 (67.8)	370 (68.8)	346 (66.0)
Professional qualification	no education	21 (4.17)	21 (3.9)	16 (3.1)
	Vocational training	216 (42.94)	224 (41.6)	248 (47.3)
	Business administrator	37 (7.36)	45 (8.4)	47 (9.0)
	Bachelor degree	103 (20.48)	94 (17.5)	77 (14.7)
	Master degree (or equal)*	121 (24.06)	142 (26.4)	127 (24.2)
	PhD	5 (0.99)	12 (2.2)	9 (1.7)

⁎ The numbers are high since until around 2010 in Germany there was no bachelor degree, but only a degree equivalent to a master degree. In the surveys, school education refers to the highest level of formal schooling completed, excluding any post-secondary education such as university degrees. Professional qualifications include additional educational achievements beyond school education, such as vocational training and higher education degrees (e.g., Bachelor, Master, PhD degrees). This distinction allows for a more nuanced understanding of the educational backgrounds of participants.

Table 4 provides a description of raw data capturing key variables of investment and risk behavior of different groups. The variables displayed are “becoming aware of investment opportunities”, “risk tolerance”, and “trading frequency”. In addition, Table 4 shows the number of answers to the respective variable for Neobroker users, general investors and Ex-Neobroker users. This table provides a foundational understanding of how different groups engage with and perceive investment opportunities, risk, and trading frequency, essential metrics for further research into behavioral patterns. The differences between the behavior patterns between different groups can be analyzed with a focus on how different groups react to external signals. Previous research has shown that trading app users react to short-term signals [9]. Table 4 is followed by Fig. 1 which illustrates the comparison between investment motivations of Neobroker users and general investors, providing insights into the diverse reasons behind investment decisions.

**Table 4:** Description of raw data.

Variable	Answer options	Neobroker users – dataset 1 (N = 257) Number of answers (%)	General investors – dataset 2 (N = 190) Number of answers (%)	Ex-Neobroker users – Dataset 1 (N = 113) Number of answers (%)
Becoming aware of investment opportunities	Own research	122 (47.5)	110 (57.9)	60 (53.1)
	Personal environment	84 (32.7)	44 (23.2)	39 (34.5)
	Financial news – daily press	107 (41.6)	79 (41.6)	35 (31.0)
	Stock market magazines	85 (33.1)	52 (27.4)	27 (23.9)
	Financial internet platforms	96 (37.4)	56 (29.5)	24 (21.2)
	Social media	80 (31.1)	18 (9.5)	22 (19.5)
	Trading App (e.g. suggestions)	92 (35.8)	14 (7.4)	20 (17.7)
	Other	11 (4.3)	29 (15.3)	9 (8.0)
Risk tolerance	0 – “not at all willing to take risks”	1 (0.4)	8 (4.2)	13 (11.5)
	1	3 (1.2)	7 (3.7)	8 (7.1)
	2	12 (4.7)	20 (10.5)	9 (8.0)
	3	21 (8.2)	12 (6.3)	10 (8.8)
	4	17 (6.6)	15 (7.9)	12 (10.6)
	5	69 (26.8)	35 (18.4)	19 (16.8)
	6	38 (14.8)	42 (22.1)	13 (11.5)
	7	45 (17.5)	29 (15.3)	19 (16.8)
	8	30 (11.7)	13 (6.8)	6 (5.3)
	9	6 (2.3)	5 (2.6)	2 (1.8)
	10 – “very willing to take risks”	15 (5.8)	4 (2.1)	2 (1.8)
Trading frequency	0 trades	8 (3.1)	46 (24.2)	8 (7.1)
	1 trade	55 (21.4)	60 (31.6)	27 (23.9)
	2-3 trades	63 (24.5)	50 (26.3)	37 (32.4)
	4-5 trades	48 (18.7)	13 (6.8)	21 (18.6)
	6-10 trades	41 (16.0)	14 (7.4)	8 (7.1)
	11-20 trades	25 (9.7)	6 (3.2)	5 (4.4)
	21-30 trades	10 (3.9)	0 (0.0)	4 (3.5)
	31-50 trades	5 (1.9)	1 (0.5)	2 (1.8)
	51-100 trades	2 (0.9)	0 (0.0)	1 (0.9)
	> 100 trades	0 (0.0)	0 (0.0)	0 (0.0)

**Note:** Multiple answers were possible for “becoming aware of investment opportunities”.

**Note:** The numbers displayed for the Ex-Neobroker users for trading frequency are consist of the numbers of Ex-Neobroker users who still invest through another broker (N = 42) and those of Ex-Neobroker users who are now Ex-Investors (N = 71).

### 3.1 Measuring of variables [1,6,7]

1. **Risk tolerance**: Participants were asked to rate their willingness to take risks on a scale from 0 to 10, where 0 signifies “not at all willing to take risks” and 10 denotes “very willing to take risks”. Previous research has shown that financial decision-making and the frequency of stock trading are influenced by attitudes toward risk among other aspects [8]. This raises the question for future research on whether risk tolerance of Neobroker users influences their investment behavior, particularly in comparison to other investor groups. In addition, the risk preferences of online-investors shape investment behavior [12]. This suggests that Neobroker users, as a special group of investors within the overall online investor group, could also be influenced by their risk tolerance in their investment behavior. The variable of risk tolerance enables a longitudinal investigation of the influence of risk tolerance on investment behavior.
2. **Financial Literacy**: The financial literacy of the participants was assessed through their responses to three fundamental questions: 1. “What is meant by the bid-ask spread?”; 2. “How do Neobrokers earn money?”; 3. “How does the value of a fixed-rate bond change when interest rates rise?”. The financial literacy score can be determined based on the number of correct answers, with one point awarded for each correct response, resulting in a score ranging from 0 to 3.
3. **Trading frequency**: To measure trading frequency, participants reported the average number of trades they execute, executed in the past or plan to execute per month, excluding automatically executed saving plans. The variable “trading frequency” derives from the average number of monthly, manually executed trades by the respective group under consideration.
4. **Annual (non-risk adjusted) return**: Participants were asked to report their average annual return, which was not adjusted for risk. A sample bias could appear to the annual (non-risk adjusted) annual return, as participants from the first survey with a negative return might not participate in the second or third survey [8].
5. **Financial products owned**: Survey participants self-reported their ownership of various financial products, including single stocks, broad ETFs, special ETFs, funds, derivatives, cryptocurrencies, and ETCs. From these answers a score for the number of different financial products owned by participants can be created. This variable does not aim to display the diversification within one specific product, but rather to show how man different products are owned.
6. **Risk assessment of financial products**: Participant assigned a risk level to each financial product on a scale from 1 to 4, where 1 signifies “low-risk”, 2 meaning “rather low-risk”, 3 corresponds to “rather high-risk”, and 4 indicates “high-risk”. The assessed financial products included single stocks, broad ETFs, special ETFs, funds, derivatives, cryptocurrencies, and ETCs. The risk assessment of financial products might possibly change due to trading app use over time or due to the stop of using a trading app during survey waves. The described score which can be built with the datasets, enable to investigate the impact of different situations regarding investing with trading apps on the risk assessment of financial products as well as a possible connection between the number of different financial products owned and the risk assessment of those products.
7. **Importance of low trading fees**: Participants were asked about their assessment of the importance of low trading fees for their broker choice. This variable spans from 1 to 4 with 1 meaning “unimportant”, 2 representing “less important”, 3 meaning “rather important”, and 4 standing for “very important”. Previous research proposed the question how free commission free trading, as it is currently possible when investing through trading apps, is [11]. This variable allows to identify the impact of low fees on the behavior of trading app users and to answer the question of whether low trading fees are more important for Neobroker users than for other groups (e.g. general investors).

## 4 Experimental Design, Materials and Methods

**Survey Design and Execution**: In collaboration with a company conducting representative surveys in Germany, the research team designed and executed a panel survey to gather comprehensive data on Neobroker usage and its influence on investment behaviors among German Neobroker users in general. This means that other than previous studies, this study focuses on Neobroker usage in general which means across various trading apps. Conducted in three waves, the survey enables in-depth longitudinal analysis. The questionnaires were designed by the research team, while the surveys were conducted by the survey company.

**Sampling Methodology**: The survey company implemented a stratified sampling method to develop a panel representative of key subsets of the German adult population. Emphasis was placed on specific demographic attributes such as age, gender, and financial product experience. A multistage probability sampling approach was employed, ensuring extensive population coverage and representativeness within target demographics.

**Fig. 2:**
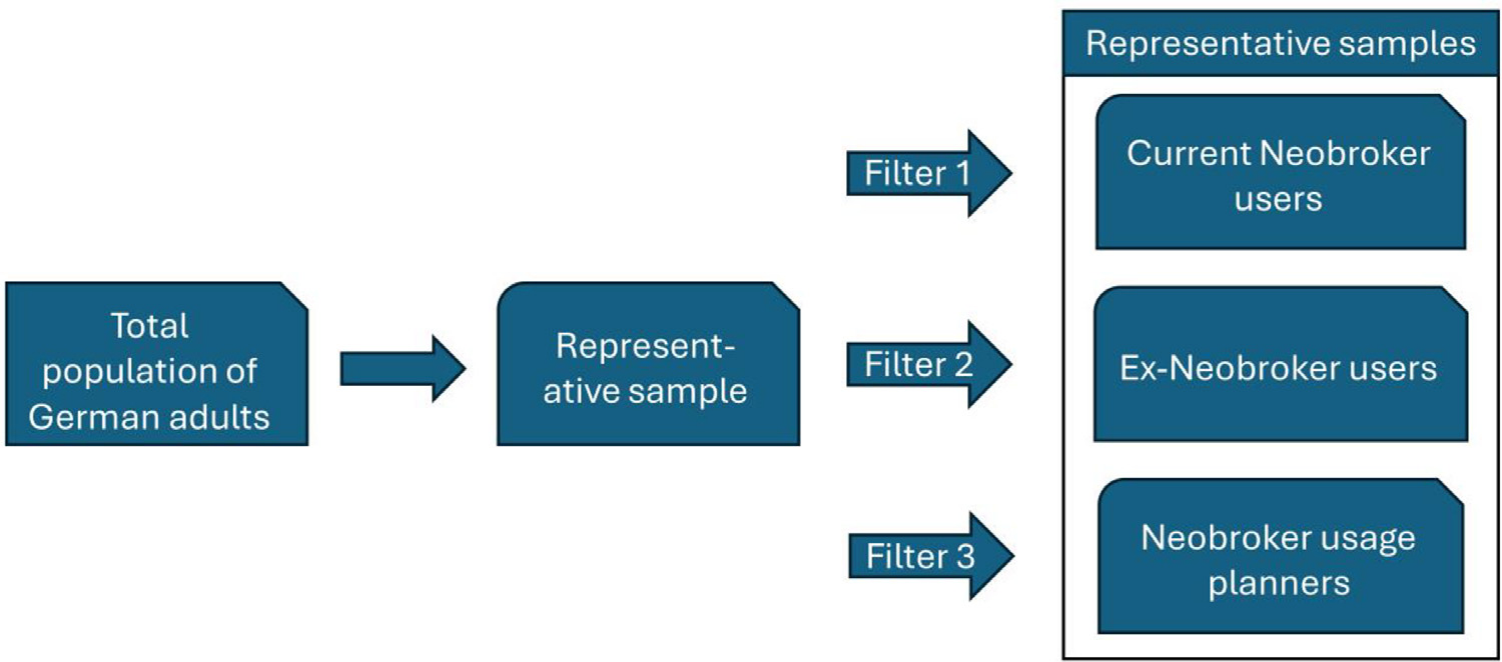
Logic of the data collection of the first dataset.

Subsamples within each wave were constructed to be representative of their respective groups, such as current Neobroker users, Ex-Neobroker users, individuals intending to use Neobrokers, and general online investors. This approach enhanced the validity and reliability of the insights derived from these cohorts. The data collection process for each wave is illustrated in Fig. 2, depicting the data collection logic for the first dataset.

**Participant Recruitment and data methodology**: In each survey, participants were recruited for each group until the predetermined target number was achieved. For the first survey, it was designed to conclude once a minimum of 250 Neobroker users, 110 former Neobroker users, and 125 prospective Neobroker users had participated. The methodology of the data collection of each survey is illustrated in Fig. 2. The company that conducted the surveys selected a group of German individuals that is representative of the German population. To ensure participants were accurately categorized into one of the target groups, filter questions were employed in the respective questionnaire. These filter questions delineated subsets such as Neobroker users, Neobroker usage planners, and former users, resulting in specific subset samples that are representative of their respective population segments in Germany.

For dataset 1, the filter questions illustrated in Fig. 2 are:

• Filter 1: “Do you currently use a trading app?”
• Filter 2: “Have you used a trading app in the past and now stopped using it?”
• Filter 3: “Are you currently planning to use a trading app in the future?”.

For dataset 2, the target groups were Neobroker users, general investors, and Neobroker usage planners. The changed filter questions reflects this adjustment:

• Filter 1: “Do you currently use a trading app?”
• Filter 2: “Do you currently invest through another broker than a Neobroker?”
• Filter 3: “Are you currently planning to use a trading app in the future?”

For dataset 3, the focus shifted to partly repeatedly surveyed Neobroker users and general investors. Hence, the filter questions were:

• Filter 1: “Do you currently use a trading app?”
• Filter 2: “Do you currently invest through another broker than a Neobroker?”

The representativeness of each subset within the datasets ensures that while the datasets do not collectively represent the entire German population, they provide representative insights into specific user groups.

**Questionnaire development**: The questionnaires were carefully developed based on established literature. They included standardized financial literacy questions [3] and measurements of financial literacy [4,5] and specific questions pertinent to Neobroker operations, such as questions related to revenue generations like Payment for Order Flow and Order Fees. A pre-study conducted by one of the researchers informed and refined the questions to mirror realistic scenarios faced by Neobroker users.

**Data collection and quality assurance**: Data collection was performed via digital surveys, administered by the survey company to maximize response rates. Every participant received compensation compliant with ethical research standards. Quality control was ensured through control questions to screen for inattentive or random responses, with low-quality responses being excluded to maintain data integrity.

**Data processing**: Data processing and analysis were conducted using SPSS, which facilitated comprehensive statistical examination and trend interpretation across the datasets.

By ensuring a diverse and representative sample in each wave, the datasets successfully capture a broad view of behavior across various groups, thereby strengthening the validity of the conclusions which can be drawn from the datasets.

## Limitations

The primary limitation encountered is panel mortality, which resulted from varying participant engagement and retention across survey waves. Despite efforts to maintain a consistent methodology and set participation criteria, some participant dropout did occur. Dropouts among Neobroker users in later waves were compensated by recruiting new users from the panel to ensure sample continuity. Additionally, the data on annual (non-risk adjusted) returns are self-reported, which may introduce bias due to social expectations and should be interpreted with caution. While sampling methodology ensured that each subset within the datasets is representative of its specific target group (e.g., current Neobroker users, former Neobroker users, and general investors), it is important to note that the combined datasets do not collectively represent the entire German population. This limitation should be considered when using the data and interpreting findings, as the results are specifically tailored to the behaviors and characteristics of the identified subgroups rather than the broader population.

## Ethics Statement

The authors confirm that all participants provided informed consent to the survey company prior to data collection. No personally identifiable information was recorded, and all data were fully anonymized to protect participant privacy. The study adhered to ethical research practices, and no formal ethical committee approval was required under the regulations applicable to this work.

## Credit Author Statement

**Jonas Freibauer**: Conceptualization, Validation, Data curation, Writing – Original Draft, Writing – Review & Editing, Visualization; Project administration; **Marc Oliver Rieger**: Conceptualization, Writing – Review & Editing, Funding acquisition; **Silja Grawert**: Conceptualization, Writing – Review & Editing, Funding acquisition.

## Data Availability

University of TrierNeobroker data (Original data)
